# *Culicoides s*pecies composition and molecular identification of host blood meals at two zoos in the UK

**DOI:** 10.1186/s13071-020-04018-0

**Published:** 2020-03-16

**Authors:** Marion E. England, Paul Pearce-Kelly, Victor A. Brugman, Simon King, Simon Gubbins, Fiona Sach, Christopher J. Sanders, Nic J. Masters, Eric Denison, Simon Carpenter

**Affiliations:** 1 The Pirbright Institute, Ash Road, Woking, Surrey GU24 0NF UK; 2 Zoological Society of London, Outer Circle, Regent’s Park, London, NW1 4BJ UK; 3 Department of Disease Control, London School of Hygiene and Tropical Medicine, Keppel St, London, WC1E 7HT UK

**Keywords:** *Culicoides*, Bluetongue virus, Arbovirus, Zoological garden, Vector-borne disease

## Abstract

**Background:**

*Culicoides* biting midges are biological vectors of arboviruses including bluetongue virus (BTV), Schmallenberg virus (SBV) and African horse sickness virus (AHSV). Zoos are home to a wide range of ‘at risk’ exotic and native species of animals. These animals have a high value both in monetary terms, conservation significance and breeding potential. To understand the risk these viruses pose to zoo animals, it is necessary to characterise the *Culicoides* fauna at zoos and determine which potential vector species are feeding on which hosts.

**Methods:**

Light-suction traps were used at two UK zoos: the Zoological Society of London (ZSL) London Zoo (LZ) and ZSL Whipsnade Zoo (WZ). Traps were run one night each week from June 2014 to June 2015. *Culicoides* were morphologically identified to the species level and any blood-fed *Culicoides* were processed for blood-meal analysis. DNA from blood meals was extracted and amplified using previously published primers. Sequencing was then carried out to determine the host species.

**Results:**

A total of 11,648 *Culicoides* were trapped and identified (*n* = 5880 from ZSL WZ; *n* = 5768 from ZSL LZ), constituting 25 different species. The six putative vectors of BTV, SBV and AHSV in northern Europe were found at both zoos and made up the majority of the total catch (*n* = 10,701). A total of 31 host sequences were obtained from blood-fed *Culicoides. Culicoides obsoletus*/*C. scoticus*, *Culicoides dewulfi*, *Culicoides parroti* and *Culicoides punctatus* were found to be biting a wide range of mammals including Bactrian camels, Indian rhinoceros, Asian elephants and humans, with *Culicoides obsoletus/C. scoticus* also biting Darwin’s rhea. The bird-biting species, *Culicoides achrayi*, was found to be feeding on blackbirds, blue tits, magpies and carrion crows.

**Conclusions:**

To our knowledge, this is the first study to directly confirm blood-feeding of *Culicoides* on exotic zoo animals in the UK and shows that they are able to utilise a wide range of exotic as well as native host species. Due to the susceptibility of some zoo animals to *Culicoides*-borne arboviruses, this study demonstrates that in the event of an outbreak of one of these viruses in the UK, preventative and mitigating measures would need to be taken.
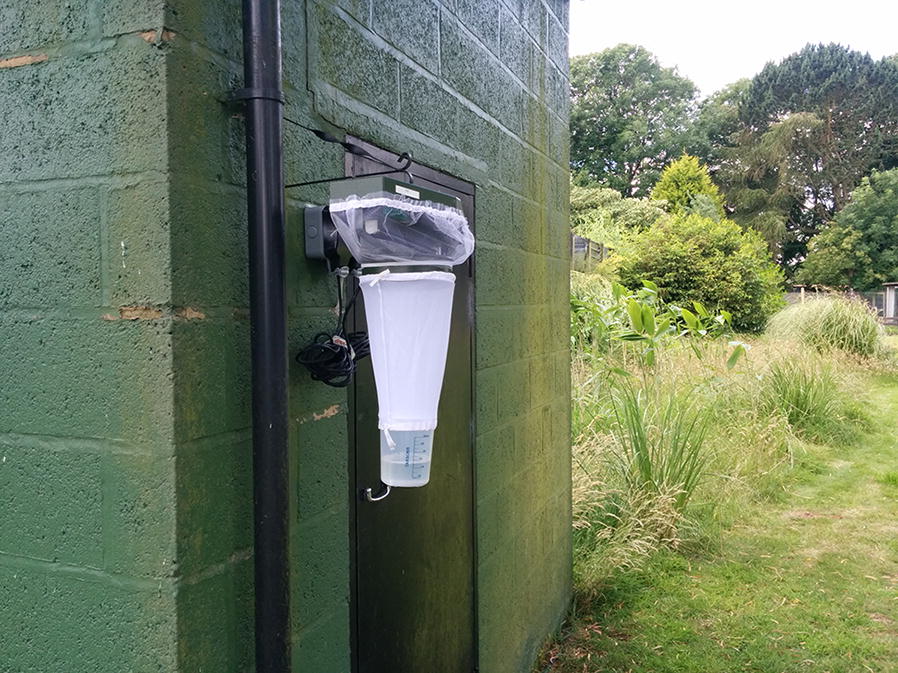

## Background

*Culicoides* biting midges (Diptera: Ceratopogonidae) transmit economically important arboviruses (arthropod-borne viruses), including bluetongue virus (BTV), Schmallenberg virus (SBV) and African horse sickness virus (AHSV) [1]. In the past two decades, BTV and SBV have inflicted unprecedented epidemics of disease in northern Europe, where there were no previous records of *Culicoides*-borne virus incursion, with major impacts on the health, productivity and trade of susceptible ruminant livestock hosts (cattle, sheep and goats) [2, 3]. AHSV is also viewed as a threat to equine hosts in Europe, although the degree to which sustained transmission could occur in northern Europe in the absence of widespread donkey or zebra reservoir hosts is unclear [4, 5].

In addition to livestock and companion animals, a range of wildlife can also become infected with BTV, SBV and AHSV, including species only distantly related to primary livestock hosts. In North America, outbreaks of both BTV and epizootic haemorrhagic disease virus (EHDV) cause severe clinical disease in cervids and impact on farming of the white-tailed deer (*Odocoileus virginianus*) [6, 7]. During the recent outbreaks of BTV and SBV in northern Europe, the potential for cervids to act as a reservoir host for these viruses was also considered, although this potential mechanism of virus persistence and re-emergence remains poorly understood [8–10].

Antibodies to BTV, SBV or AHSV have also been found in a wide range of other mammals including dogs (*Canis lupis familiaris*), wild dogs (*Lycaon pictus*), jackals (*Canis* spp.), lions (*Panthera leo*), spotted hyenas (*Crocuta crocuta*), black bears (*Ursus americanus*), African elephants (*Loxodonta africana*), white rhinoceros (*Ceratotherium simum*) and a range of antelope species (Bovidae) [11–15]. Herbivorous species are thought to become infected with BTV primarily through biological transmission by *Culicoides* spp., while infection of carnivores is ascribed to feeding on meat from infected mammals. However, specifying these transmission routes is challenging and evidence is largely anecdotal [11, 14]. *Culicoides* spp. have been shown to feed on domestic dogs [16] and AHSV has been detected in domestic dogs with no history of ingestion of horse meat [17]. For BTV, the potential for animals of these species to develop a transmissible viraemia remains unclear in all species with the exception of deer. Disease outcomes of infection in wildlife also remain unpredictable for all three viruses, particularly in areas of emergence or re-emergence [11, 12].

In the UK, seroconversion to SBV was detected in a range of exotic animals screened at the Zoological Society of London (ZSL) London Zoo, ZSL Whipsnade Zoo and Chester Zoo using a cELISA and positive samples were confirmed and quantified using a plaque reduction neutralisation test [18]. Species testing positive for exposure to SBV on both assay systems included Asian elephants (*Elephas maximus*), reticulated giraffes (*Giraffa camelopardalis reticulata*), red river hogs (*Potamochoerus porcus*), deer (hog deer, *Axis porcinus*; reindeer, *Rangifer tardinus*), antelopes (greater kudu, *Tragelaphus strepsiceros*; blackbuck, *Antelope cervicapra*) and bovids (yak, *Bos grunniens*; gaur, *Bos gaurus*). No clinical disease was reported in any of these hosts, but some UK zoos have previously carried out precautionary vaccination of high-value animals against BTV-8 (T. Woodfine, personal communication., NM, unpublished data). At present for BTV all post-import testing of susceptible exotic ruminants (restricted to the Cervidae, Camelidae, Giraffidae, Antilocapridae and Bovidae) and movements are facilitated on a case-by-case basis through bilateral agreements based on Article 8.1(b) of Commission Regulation (EC) No. 1266/2007. Equine hosts susceptible to AHSV are also subject to stringent pre- and post-movement testing procedures. Infection with SBV is not routinely examined in these species.

Globally, just three studies have investigated *Culicoides* populations in zoos. One study carried out trapping for *Culicoides* over a two-year period at the National Zoological Gardens in Pretoria, South Africa using Onderstepoort Veterinary Institute (OVI) light-suction traps [19]. These detected the presence and high abundance of the main afrotropical vector of BTV and AHSV, *Culicoides imicola* Kieffer, 1913, alongside 36 other species known to utilise mammalian and avian hosts [19]. In the USA, a study used Centre for Disease Control (CDC) traps baited with ultraviolet light (model 1212) and ABC traps baited with incandescent light, to survey *Culicoides* populations in two zoos in South Carolina [20]. These surveys detected 16 species of *Culicoides* including putative vectors of BTV.

In the UK, trapping for *Culicoides* was carried out at Chester Zoo as part of a preliminary experiment in June 2008, at five sites over four consecutive nights using OVI traps [21]. Over 35,000 *Culicoides* were collected, and 25 species recorded, including all species implicated in the recent outbreaks of SBV and BTV in northern Europe [1, 3]. Interestingly, large catches greater than 1000 individuals in a single trap night were made from within enclosures containing white rhinoceros and zebra, indicating a high local abundance of potential vector species [21]. However, there was no attempt to examine feeding history or preference of the collected *Culicoides*.

Following outbreaks of *Culicoides*-borne arboviruses across Europe, a series of studies across the region have used identification of blood meals *via* molecular assays to define host range [22]. These studies have demonstrated that while *Culicoides* usually exhibit a preference for either avian or mammalian hosts, they blood-feed on a wide variety of species within these classes. Within those species that feed on mammals, those that have been implicated as primary vectors in transmission of SBV and BTV demonstrate broad host range, including ruminants, equids, camelids, lagomorphs and rodents [23, 24]. This is despite significant variation in the degree to which these are reliant on livestock for larval development sites (*Culicoides obsoletus* (Meigen, 1818) and *Culicoides scoticus* Downes & Kettle, 1952 develop in a wide range of organically enriched substrates while in contrast *Culicoides dewulfi* Goetghebuer, 1936 and *Culicoides chiopterus* (Meigen, 1830) develop in animal dung) [25, 26]. Within the UK, there have been very few studies that have carried out blood-meal analysis of *Culicoides.* One study confirmed that potential UK *Culicoides* vector species of AHSV were blood-feeding on horses, proving a direct host-vector interaction [27]. Another study found that *Culicoides impunctatus* Goetghebuer, 1920, a species that is generally considered to have a very minor or no role in disease transmission, had fed on cows, sheep, deer and humans in Scotland [28]. It is important to establish host preferences of vector species and the extent of opportunistic biting behaviour as these have implications for disease spread and may affect disease dynamics in an outbreak scenario [5, 29].

In this study we used DNA sequencing of a mitochondrial-derived marker to directly link *Culicoides* populations with blood-feeding on exotic animals in zoological gardens for the first time. Additionally, we examined the seasonality of adult flying populations at these sites in order to understand how transmission risk fluctuates across seasons within these environments. We compared these results with standard surveillance schemes, with particular reference to the seasonal vector-free period (SVFP) as defined by the collection of < 5 pigmented female vector *Culicoides* [30]. These data are important for understanding and quantifying the risk of *Culicoides-*borne viruses to susceptible, valuable and in some cases, endangered, zoo animals. They also provide insight into the utilisation of hosts to which these species of *Culicoides* have not been exposed previously.

## Methods

### Trapping and identification of *Culicoides*

**Fig. 1:**
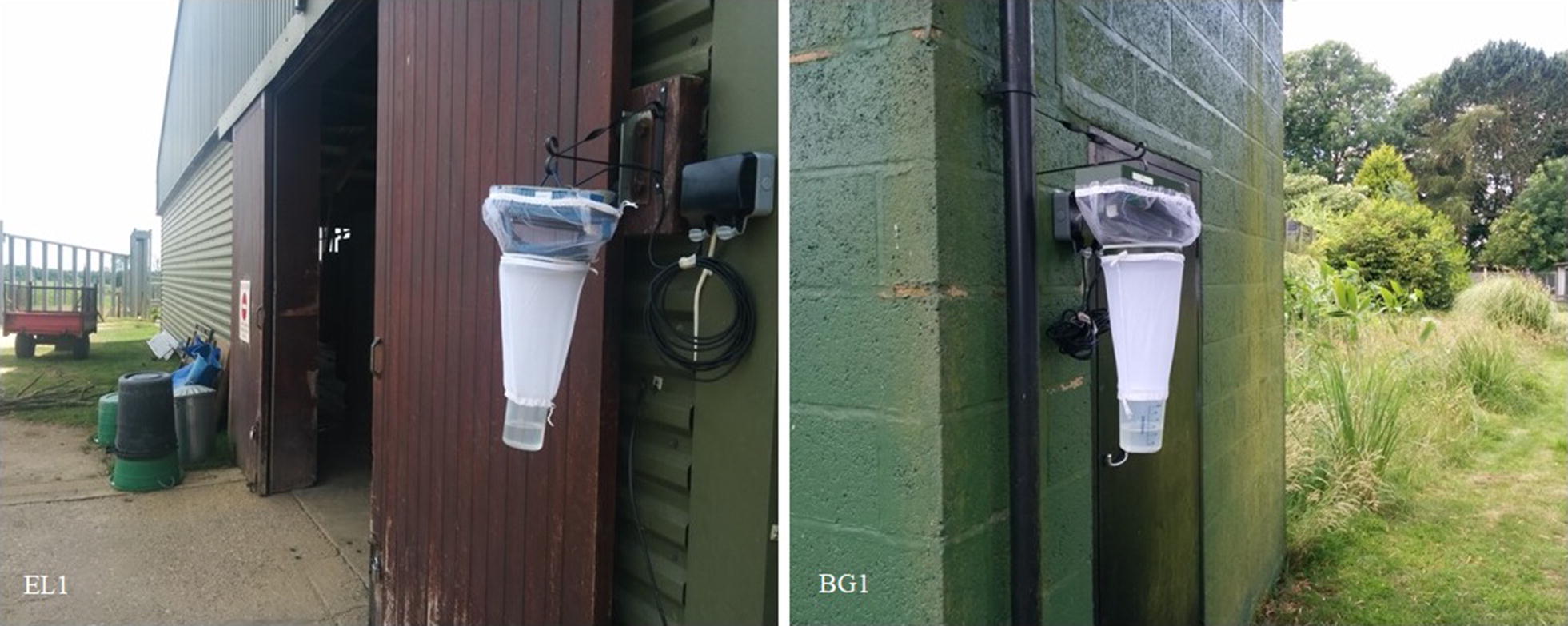
Two OVI light-suction traps located at ZSL Whipsnade Zoo. Trap EL1 located next to the elephant enclosure and Trap BG1 located in the bird garden

Onderstepoort Veterinary Institute (OVI) 220V light-suction traps were used to monitor *Culicoides* populations at two zoological gardens using standard surveillance approaches (Fig. 1) [31]. The zoos chosen were ZSL London Zoo (ZSL LZ) and ZSL Whipsnade Zoo (ZSL WZ) sites. ZSL LZ (51°32′6.2268′′N, 0°9′13.0824′′W) is located in an urban setting on the edge of Regent’s Park in central London. In contrast, ZSL WZ (51°50′39.1236′′N, 0°32′27.8772′′W) is located in a rural area surrounded by countryside, at a higher altitude (216.72 m above sea level compared to 35.88 m above sea level at ZSL LZ). The vegetation within the exhibits at ZSL LZ is mostly made up of exotic species of shrubs and trees, with large areas of paving and small lawns in between exhibits. The zoo is adjacent to Regent’s Park, characterised by lawns with native trees and hedgerows. There are many large open paddocks with native trees at ZSL WZ, with exotic planting close to animal housing and in smaller exhibits. ZSL LZ holds a collection of 60 different species of mammal, 97 species of bird, 49 species of reptile and 20 species of amphibian, constituting 2125 individuals excluding fish and invertebrates while ZSL WZ holds a collection of 56 species of mammal, 64 species of bird and 17 species of reptile, constituting 1364 individuals excluding fish and invertebrates [32].

**Fig. 2:**
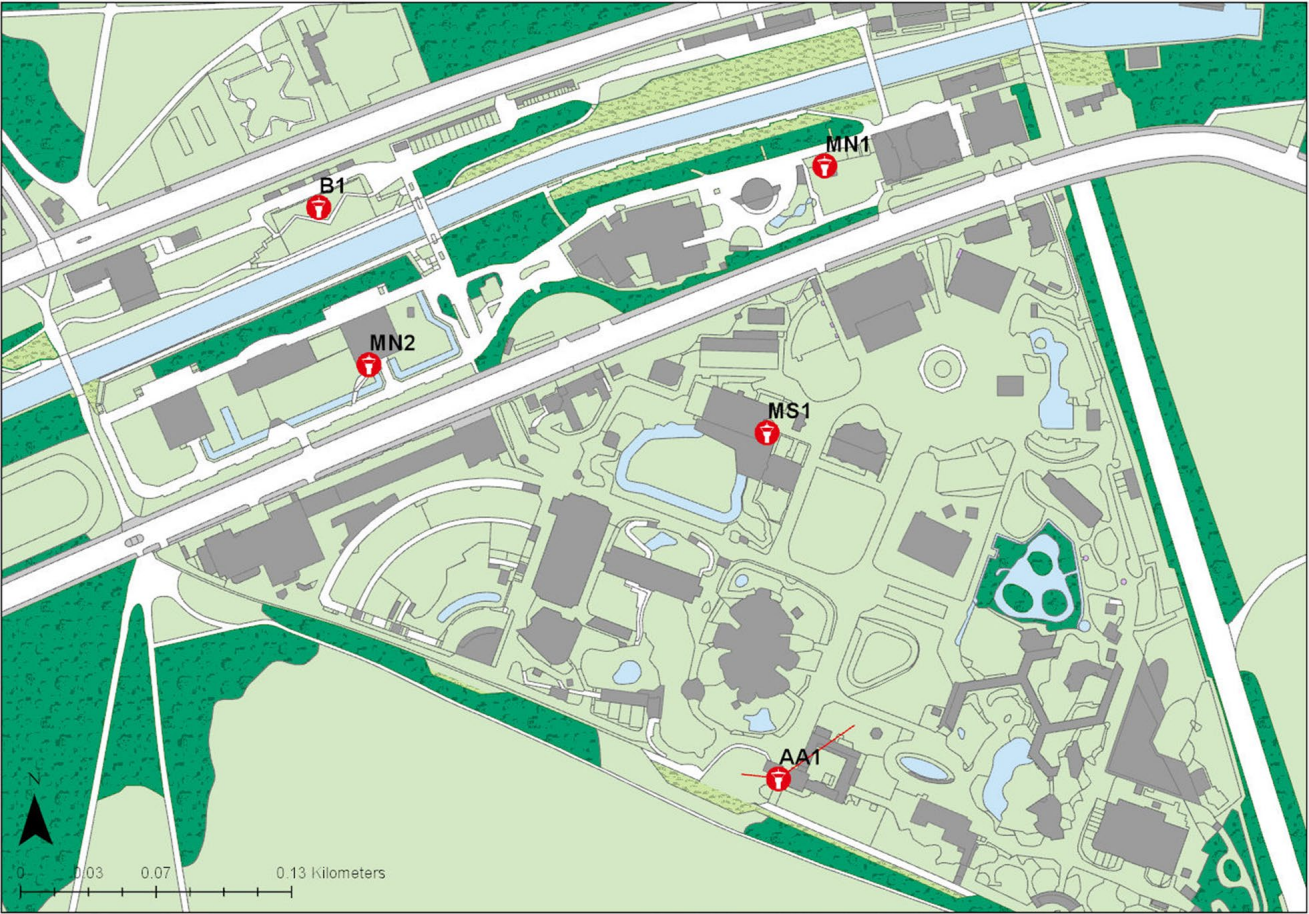
Map of ZSL London Zoo showing trap locations. The red lines indicate the distance between the traps where blood-fed *Culicoides* were caught and the location of the respective host (if known)

**Fig. 3:**
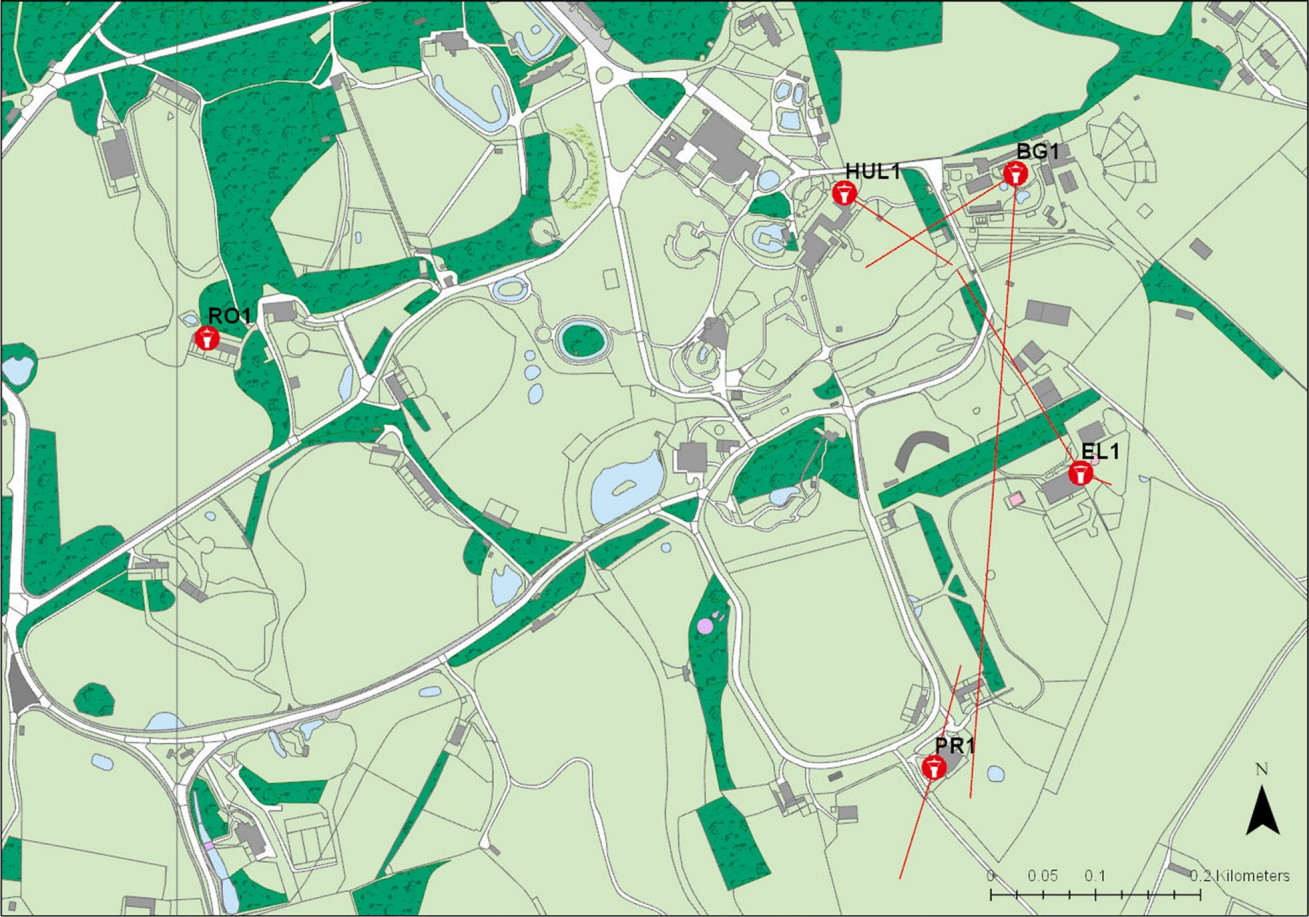
Map of ZSL Whipsnade Zoo showing trap locations. The red lines indicate the distance between the traps where blood-fed *Culicoides* were caught and the location of the respective host (if known)

Five trap locations were used at each zoo, with site selection based on targeting a range of host species, including those susceptible to bluetongue and clinical signs of SBV (Figs. 2, 3). Trapping was conducted from June 2014 to June 2015. OVI 8w light-suction traps were run overnight for one night each week by a volunteer and collections made into water with a drop of detergent were sieved and transferred into 70% ethanol for identification and storage. *Culicoides* were morphologically identified to species level under a dissecting microscope using published keys [33–35]. Females of *C. obsoletus* and *C. scoticus* were grouped together as *C. obsoletus* complex. Female *Culicoides* were further classified as unpigmented, pigmented, gravid or blood-fed based on the morphology of their abdomen [36].

The Corine Land Cover (CLC) 2018 map (available from https://land.copernicus.eu/) was used to compare the land cover at each site. The percentage of each land cover class was extracted from under a buffer zone of radius 3125 m from the centre of each zoo, using ArcGIS Pro 2.3.1 (ESRI, Redlands, CA, USA). The radius of the buffer zone was set according to the maximum dispersal distance identified by a previous study on *Culicoides* in the south of England [37]. The percentage of each Corine land cover class that fell within the buffer zones is summarised in Table 1. ZSL LZ and the surrounding area is dominated by urban fabric (86.4%), whereas ZSL WZ and the surrounding area is dominated by arable land and pastures (82.2%), with urban fabric constituting just 3.1% of the land cover.

**Table 1:** Percentage of each land cover classification at ZSL London Zoo and ZSL Whipsnade Zoo

London Zoo		Whipsnade Zoo	
Land cover class	% cover	Land cover class	% cover
Discontinuous urban fabric	55.9	Non-irrigated arable land	62.3
Continuous urban fabric	30.4	Pastures	19.9
Green urban areas	7.7	Sport and leisure facilities	7.5
Sport and leisure facilities	2.9	Coniferous forest	3.7
Road and rail networks	2.0	Discontinuous urban fabric	3.1
Industrial/commercial units	1.0	Mineral extraction sites	1.5
		Agriculture, with natural vegetation	1.4
		Broad-leaved forest	0.5

### Blood-meal molecular analysis

After species identification, *Culicoides* that contained blood were transferred into individual 1.5 ml Eppendorf tubes containing 200 µl phosphate-buffered saline (PBS) and homogenised with a pellet pestle for 30 s. Following the addition of 20 µl proteinase K and 200 µl buffer AL, each sample was incubated at 56 °C for 30 min. Host DNA was then extracted from the sample using the Qiagen DNeasy Blood and Tissue Kit (Qiagen, Manchester, UK), following the manufacturer’s instructions (see Additional file 1: Text S1). Samples were stored at − 20 °C until further analysis.

A 685 bp region of the cytochrome *c* oxidase subunit 1 (*cox*1) gene was targeted using a combination of existing primers [38]. The polymerase chain reaction (PCR) was performed in a final volume of 25 µl comprising 2.5 µl of PCR buffer, 0.75 µl of 1.5 mM MgCl_2_, 0.5 µl of 200 µM dNTP, 0.25 µl of 0.1 µM primers VF1_t1, VF1d_t1, VR1_t1 and VR1d_t1, 0.5 µl of 0.2 µM primers VF1i_t1 and VR1i_t1, 5 µg bovine serum albumin, 0.1 µl of 1 U Platinum Taq DNA polymerase (Invitrogen, Paisley, UK), 13.9 µl of nuclease-free water and 5 µl extract. The PCR cycling conditions were an initial denaturation of 94 °C for 2 min, followed by 40 cycles of (i) 94 °C for 30 s; (ii) 54 °C for 30 s; and (iii) 72 °C for 1 min. A final elongation step of 72 °C for 10 min was used. PCR products were visualised on a 1.5% agarose gel and samples of the correct size were purified using the Illustra GFX PCR purification kit (GE Healthcare, Amersham, UK), following the manufacturer’s instructions (Additional file 2: Text S2). Purified PCR products were sequenced bi-directionally using M13 primers, by Source Biosciences (Cambridge, UK). Sequence electropherograms were checked manually and assembled using SeqMan Pro v14 (DNAStar, Madison, USA). DNA sequences derived from blood meals were compared against all available sequences on GenBank using BLAST and assigned to a host vertebrate species with a match of > 98% [39].

### Temperature data

Temperature data for 2014 and 2015 were obtained from the UK Climate Projections (UKCP09) gridded observation datasets (Additional file 3: Figure S1). These cover the UK at 5 × 5 km resolution with the data for each zoo extracted for the grid square in which it is located.

### Statistical analysis

The daily trap catches for each species/group were analysed using generalised linear models with a log link function and either a Poisson or negative binomial distribution. In the model for each species, the expected catch (*µ*_*jk*_) for the *j*th collection at trap *k* (taken on day t_*jk*_) was given by:

$$ \log (\mu_{jk} ) = a_{k} + \sum\limits_{n = 1}^{2} {b_{1n} \sin \left( {\frac{2n\pi }{365}t_{jk} } \right) + b_{2n} \sin \left( {\frac{2n\pi }{365}t_{jk} } \right)} + c_{{y_{jk} }} + dT_{k} (t_{jk} ), $$

where *a*_*k*_ is the baseline catch for trap *k*, the summation describes seasonality in the *Culicoides* population [using sine and cosine functions with periods of 12 (*n* = 1) and 6 (*n* = 2) months], *c*_*y*_ captures the difference in catch between years and *T*_*k*_(*t*) is the daily mean temperature for trap *k* on day *t*.

Model construction proceeded by stepwise deletion of non-significant (*P* > 0.05) terms (as judged by a likelihood ratio test), starting from a model including sine and cosine functions with twelve and six month periods (i.e. describing seasonality), daily mean temperature, year (2014 or 2015) and trap location. The statistical models were implemented using the *MASS* package [40] in R (version 3.4.3) [41]. Models were fitted to data for total *Culicoides*, *C. obsoletus/C. scoticus* females, *C. chiopterus* females, *C. pulicaris* females and *C. punctatus* females. However, there were too few females of *C. dewulfi* or males of any species collected to allow robust models to be fitted.

## Results

A total of 11,648 *Culicoides* (5880 from WZ and 5768 from LZ) were collected and identified (Additional file 4: Dataset S1) from a total of 280 trap catches (118 from WZ and 162 from LZ, Table 2). Twenty species of *Culicoides* were caught at ZSL LZ and 18 different species of *Culicoides* were caught at ZSL WZ. The majority of specimens caught (92%, *n* = 10,701) were of the species described as putative vectors of BTV and SBV in northern Europe. i.e. members of the subgenus *Avaritia* (*C. obsoletus*, *C. scoticus*, *C. dewulfi* and *C. chiopterus*), *C. pulicaris* and *C. punctatus*. Of these, *C. obsoletus* and *C. scoticus* alone constituted 71.5% of collections (Table 3). A total of five *Culicoides* (0.02%) could not be identified due to damage.

**Table 2:** Trapping sites used at London and Whipsnade Zoological Gardens and associated *Culicoides* collections

Zoo	Trap site	Animals close to trap	Habitat	No. of species trapped	Max catch in single night	Total
LZ	AA1	Camels, llamas, alpacas, pig, goats	Yard	17	461	3557
	B1	Mixed bird species	Aviary	12	24	156
	MN1	Reindeer	Paddock	6	55	116
	MN2	Giraffe	Paddock	12	1584	1830
	MS1	Primates	Yard	12	35	109
WZ	BG1	Mixed bird species	Lawn	11	106	224
	HUL1	Goats, sheep, alpacas, llamas, donkeys, pigs and rabbits	Yard	11	172	455
	PR1	Przewalski’s horse, camels, yak, and Indian rhinoceros	Paddock	13	616	1008
	EL1	Asian elephants	Yard	16	1393	3922
	RO1	Roan antelope, giraffe and Thomson’s gazelle	Paddock	12	58	271
Total						11,648

**Table 3:** Species and abundance of *Culicoides* trapped at each zoo across the sampling sites

Species	London Zoo (% total)	Whipsnade Zoo (% total)	Total (% total)
*C. obsoletus/C. scoticus* (female)	3972 (68.9)	4173 (71.0)	8145 (69.9)
*C. obsoletus* (male)	24 (0.4)	72 (1.2)	96 (0.8)
*C. scoticus* (male)	28 (0.5)	57 (1.0)	85 (0.7)
*C. dewulfi* (total)	138 (2.4)	268 (4.6)	406 (3.5)
*C. chiopterus* (total)	105 (1.8)	92 (1.6)	197 (1.7)
*C. pulicaris* (total)	222 (3.9)	307 (5.2)	529 (4.5)
*C. punctatus* (total)	1152 (20.0)	91 (1.5)	1243 (10.7)
Other (total)	123 (2.1)	819 (13.9)	942 (8.1)
Damaged (unidentified)	4 (0.07)	1 (0.02)	5 (0.04)
Total	5768	5880	11,648

For most trap locations at both ZSL LZ and ZSL WZ, the catches were dominated by members of the subgenus *Avaritia*. The main exception is the trap located at the Snowdon Aviary (B1) at ZSL LZ, where a wide range of species contributed to a relatively small catch. At ZSL LZ, the trap located next to the Bactrian camels (AA1) caught the most *Culicoides*, with the trap located next to the giraffe (MN2) collecting the most individuals in a single night (1584 individuals, Table 2). Traps MS1 and MN1 were not run during 2015 due to damage and, hence, collected lower numbers in total. At ZSL WZ, the trap located next to the Asian elephants (EL1) caught the most *Culicoides* and collected the most in a single night (1393 individuals, Table 2). Traps AA1 and EL1 also caught the greatest variety of species (*n* = 17 and *n* = 16, respectively, Table 2).

Female *Culicoides* were categorised as unpigmented, pigmented, gravid or blood-fed (Table 4). Unpigmented *Culicoides* constituted 63% of the total identifiable catch, with pigmented constituting 25.5%, gravid constituting 5.9% and blood-fed constituting 0.61%. A total of 571 males were trapped, constituting 4.9% of the total identifiable catch. At LZ, the seasonal vector-free period (SVFP) started on 6th November 2014, compared to 15th October 2014 at WZ. The SVFP ended on 15th April 2015 at both zoos.

**Table 4:** Number of male and age-graded female *Culicoides* caught in each trap

Zoo	Trap site	Trap catches	Females				Males	Total (% catch)
			Unpigmented	Pigmented	Gravid	Blood-fed		
London	AA1	45	2344	1116	45	7	42	3554 (61.6)
	B1	42	30	18	91	1	16	156 (2.7)
	MN1	24	83	26	4	0	3	116 (2.0)
	MN2	35	1352	383	74	0	20	1829 (31.7)
	MS1	16	58	22	21	0	8	109 (1.9)
Total		162	3867	1565	235	8	89	5764
Whipsnade	BG1	24	88	20	69	3	44	224 (3.8)
	HUL1	25	294	110	29	5	17	455 (7.7)
	PR1	19	384	276	151	10	186	1007 (17.1)
	EL1	25	2620	965	134	43	160	3922 (66.7)
	RO1	25	91	36	67	2	75	271 (4.6)
Total		118	3477	1407	450	63	482	5879
Total from both zoos		280	7344	2972	685	71	571	11,643

*Notes*: Females were classified as unpigmented, pigmented, gravid or blood-fed according to their abdomen. A further five *Culicoides* were damaged and could not be identified or age-graded

### Blood-meal analysis

**Fig. 4:**
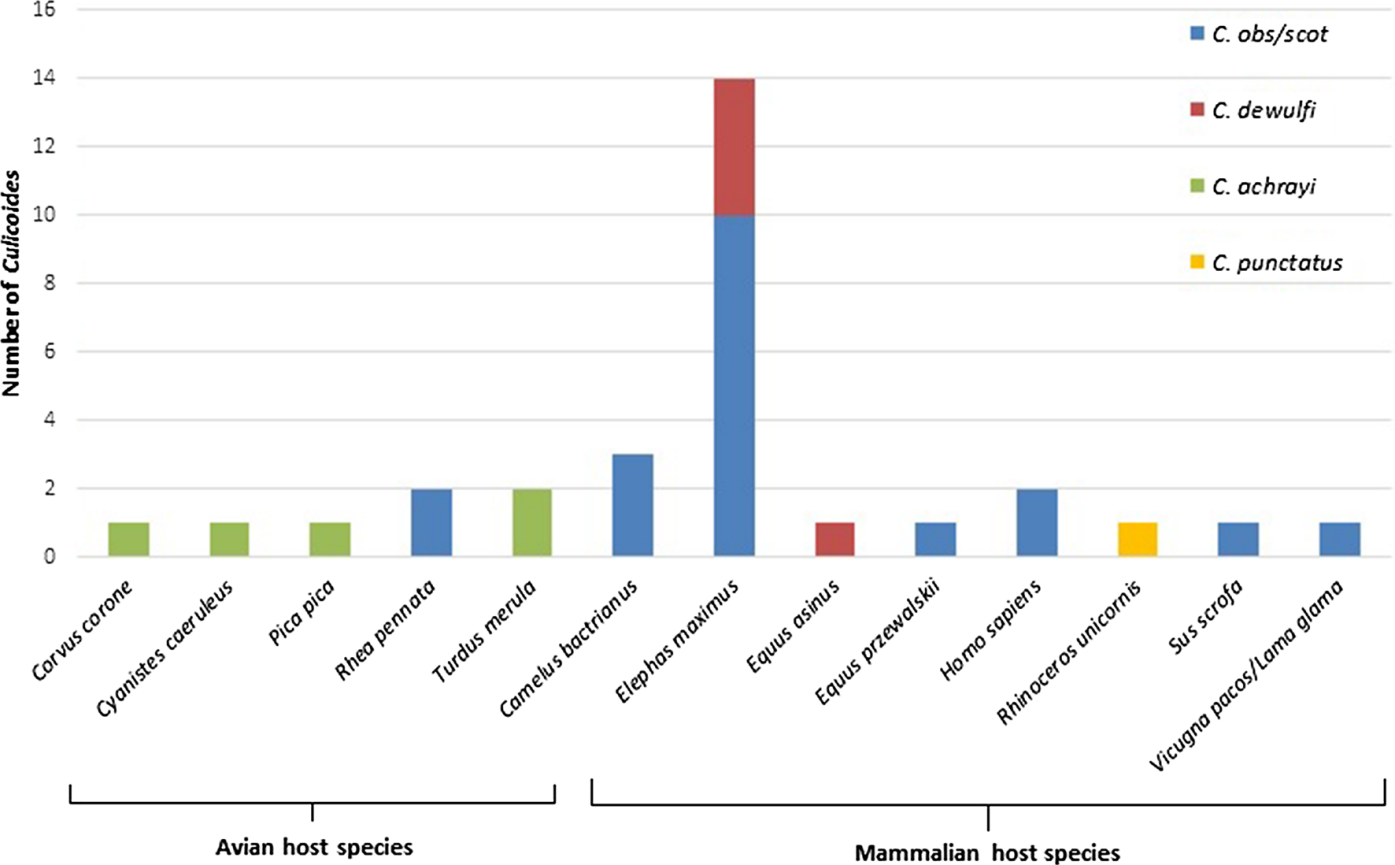
Host blood meals identified from *Culicoides* trapped at ZSL London Zoo and ZSL Whipsnade Zoo

In total, 71 *Culicoides* contained a blood meal (0.61% of total individuals caught, Additional file 5: Table S1). Of these, three blood-fed individuals were not processed for blood meal analysis as they contained only a partial blood meal at an advanced stage of digestion. Sequences which could be matched to vertebrate hosts were obtained from 31 *Culicoides* (46% of processed blood-fed individuals, Additional file 6: Table S2). Most sequences aligned to mammalian species (*n* = 24, 77%), with the remaining comprising avian species (*n* = 7, 23%). A total of 13 different host species were identified (Fig. 4). Most host blood meals identified were from exotic zoo animals (*n* = 22, 71%), demonstrating opportunistic feeding behaviour of *C. obsoletus/C. scoticus*, *C. dewulfi* and *C. punctatus*.

*Culicoides obsoletus/C. scoticus* fed on four different exotic mammalian species: Asian elephants (*Elephas maximus*), alpaca/llama (*Vicugna pacos*/*Lama glama*), Bactrian camels (*Camelus bactrianus*) and Przewalski’s horse (*Equus przewalskii*). A single blood-fed *C. punctatus* had fed on an Indian rhinoceros (*Rhinoceros unicornis*) and four *C. dewulfi* had fed on Asian elephants. Additionally, we were unable to discriminate between domestic pig (*Sus scrofa domesticus*) and wild boar (*Sus scrofa scrofa*) as the host of a blood-fed *C. obsoletus/C. scoticus*. Both wild boar and domestic pigs were present at ZSL WZ at the time of trapping. Blood meals from *Culicoides achrayi* Kettle & Lawson, 1955 were all identified as being from indigenous bird species. The only exotic bird species identified was Darwin’s rhea (*Rhea pennata*) in blood-fed *C. obsoletus/C. scoticus*. Some blood-fed *Culicoides* were collected from traps that were not close to the animal that they had fed on, suggesting some level of post-feeding dispersal by female *Culicoides* (Figs. 2, 3).

### Statistical analysis

For each species full details of model selection are provided in Additional file 7: Table S3 and the coefficients for the final models are presented in Additional file 8: Table S4; here we summarise the results across all species/groups.

There was significant (*P* < 0.001) seasonal variation in trap catches for all five species/groups analysed, with peaks in spring (May-June) and autumn (September-October) (Additional files 9: Figures S2, Additional files 10: Figure S3, Additional file 11: Figure S4, Additional file 12: Figure S5, Additional file 13: Figure S6). Specifically, the spring peak was in mid-May for total *Culicoides*, *C. obsoletus/C. scoticus* females and *C. punctatus* females, in late May for *C. chiopterus* females and in late June for *C. pulicaris* females. The autumn peak was in mid-September for total *Culicoides*, *C. obsoletus/C. scoticus* females, *C. pulicaris* females and *C. punctatus* females and in early October for *C. chiopterus* females. Abundance was greatest at the spring peak for total *Culicoides*, *C. obsoletus/C. scoticus* females, *C. pulicaris* females and *C. punctatus* females and at the autumn peak for *C. chiopterus* females. In addition, trap catches differed significantly (*P* < 0.001) between years, with around a 10-fold reduction in numbers caught in 2015 compared with 2014 for each species/group. Significantly (*P* < 0.001) higher trap catches were associated with warmer temperatures for all species/groups (Additional file 8: Table S4).

There were significant (*P* < 0.001) differences amongst traps in the numbers of *Culicoides* collected and these differences were broadly similar for all species/groups (Additional file 8: Table S4, Additional files 9: Figures S2, Additional files 10: Figure S3, Additional file 11: Figure S4, Additional file 12: Figure S5, Additional file 13: Figure S6). Significantly more *Culicoides* were caught in the trap located at the elephant enclosure (EL1) at ZSL WZ than any of the other trap locations at either zoo. Significantly more *Culicoides* were caught in the trap AA1 compared with any of the other traps at ZSL LZ, with the exception of trap MN2 that caught similar numbers to trap AA1 for total *Culicoides* and *C. obsoletus*/*C. scoticus* females. The numbers of *Culicoides* caught in the remaining traps (B1, MN1 and MS1) did not differ significantly. Comparing only traps at ZSL WZ, significantly more midges were caught in trap EL1 than any of the other traps (BG1, HUL1, PR1 and RO1). The numbers caught in these traps did not differ significantly.

## Discussion

This study has demonstrated seasonal activity of adult *Culicoides* in proximity to exotic animals in two UK zoos and provided direct evidence of blood-feeding behaviour by vectors of BTV and SBV on exotic zoo animals in northern Europe for the first time. Most blood meals identified in UK *Culicoides* species were taken from exotic animals, rather than native wildlife, demonstrating opportunistic feeding on hosts to which these species of *Culicoides* have not been previously exposed. Divergence in host selection was observed between avian (*C. achrayi*) and mammalian (*C. dewulfi*) feeders, but *C. obsoletus/C. scoticus* was found to feed on both classes of host. Further investigation is required to understand the drivers of host location involved in these observations which may include a wide range of visual, thermal and olfactory cues [22, 42].

The diversity of species of *Culicoides* collected in the two zoological gardens was similar, with 20 different species caught at ZSL LZ and 18 different species of *Culicoides* caught at ZSL WZ from a total of 46 species recorded in Britain [35]. Within these collections, five species were found only at ZSL WZ and seven species were found only at ZSL LZ. The observed difference in faunal composition of *Culicoides* at each zoo may be related to trap placement within each zoo or may reflect genuine differences in the *Culicoides* community at each zoo. Notably, all putative vectors of BTV and SBV in northern Europe were identified at both zoos. All species collected had been previously identified within the UK and the species composition at each zoo is typical of that found at livestock farms across northern Europe [31, 34, 43]. The findings are also consistent with the species composition found at Chester Zoo previously [21], where 25 species of *Culicoides* were identified. It is not known the extent to which the artificial diet of the exotic zoo animals can influence the composition of their dung and thus the suitability thereof for *Culicoides* larval development.

There have been no studies to date in zoological gardens that have identified whether *Culicoides* can adapt to breeding in the dung of exotic ruminant or equine hosts or in habitats enriched from a source that may be substantially different in constitution and microfauna compared to that from domestic livestock. Within the two zoos examined, dung is cleared from paddocks and animal houses on a daily basis. At ZSL LZ, the waste material is loaded straight into a compactor. At ZSL WZ, there is a dung pile close to the elephant enclosure where all the collected material from across the zoo is deposited. This is then removed from the site on an *ad hoc* basis (M. Shillingford, personal communication) providing a temporary abundance of potential larval development sites. The differences in the land cover between sites would suggest that ZSL WZ would support a greater diversity of *Culicoides* species and in greater abundance as a primarily rural site. Indeed, livestock are present on farms within 1 km of the zoo. This is not, however, borne out in the collections made during this study, with two more species being found at ZSL LZ than ZSL WZ and the small difference in number of individuals caught between the sites. This suggests that *Culicoides* are able to adapt to an urban landscape and colonise the small pockets of suitable habitat that are available in addition to being able to disperse significant distances to find hosts [37].

Inferring host preference within *Culicoides* is challenging and cannot be quantified without direct collections on the animals themselves which is very challenging to impose on wildlife hosts [44–46]. Within the present study, relatively clear demarcation was found in *C. achrayi* feeding solely on avian hosts, as expected from previous studies [22, 47, 48]. In a rare, direct, investigation of the blood-feeding behaviour of *Culicoides circumscriptus* Kieffer, 1918 (a bird-biting species) in Spain, this species was identified at the nests of cavity-nesting birds, where it was found to contain haemosporidian parasites [49]. Infection with these malaria-like parasites causes chronic infections in wild birds, although their impact on condition and survival remain significant, but poorly understood, including in relation to the impact of infection of exotic birds with European lineages [50, 51].

In *Culicoides* blood-feeding on mammals, there was no evidence that feeding had occurred on livestock or wildlife that were not contained within the zoo. Domestic pig and wild boar could not be separated based on the *cox*1 gene that we targeted for blood-meal analysis. At the time the study was carried out, ZSL WZ had 14 wild boar and five domestic pigs within its collection (H. Jenkins, personal communication). Therefore, it is not possible to tell if the *Culicoides* had fed on the wild boar that are in an enclosure some distance from the trap in question (HUL1) or the domestic pigs that were close to the trap. The nearest pig farm was located approximately 2 km from the trap and, therefore, may have been a source, although less likely than those animals within the zoo boundary.

*Culicoides* found to have fed on an animal species that was not held in close proximity of the trap that they were caught in, are indicated in Figs. 2 and 3 by red lines, showing the distance from host location to trap. The furthest flight documented was 600 m in the case of a *C. obsoletus/C. scoticus* female that had fed on a Przewalski’s horse and was subsequently caught in the trap located in the bird garden, BG1. The dispersal of blood-fed *Culicoides* has not previously been quantified in this way and the presence of readily identifiable and static hosts presents an ideal environment to investigate this aspect of their ecology.

Previous studies have had a higher success rate with the number of blood meals that they have been able to amplify. In this study, our success rate was approximately 50%, whilst previous studies have had up to 90% success rate [23]. This could be due to a combination of blood being partially digested by the time the *Culicoides* was trapped, DNA degradation from time spent in storage, or samples not being transferred to ethanol quickly enough following collection. Improper storage has been noted previously as a reason for lower success rates of blood meal identification [52]. Twenty-four of the 68 blood-fed specimens processed for blood meal analysis contained only partial blood meals and of these, a total of 15 failed to amplify. Some previous studies only processed fully engorged specimens, or a subset of blood-fed *Culicoides*, and as such have achieved high success rates [23, 53]. However, we processed all fully and partially blood-fed specimens, with the exception of three *C. obsoletus/C. scoticus* which contained a very small amount of blood in an advanced stage of digestion and were deemed unsuitable for analysis. There were no mixed blood meals identified.

Within the zoos, the trap that caught the most *Culicoides* was AA1 at ZSL LZ and EL1 at ZSL WZ. These traps were located next to Bactrian camels and Asian elephants, respectively. A previous study conducted at the National Zoological Gardens of South Africa, also found that the trap closest to the elephants collected the most *Culicoides* [19]. A total of 13 *Culicoides* were found to have fed on Asian elephants and four of these *Culicoides* were *C. dewulfi* (30%), with the remaining nine being *C. obsoletus/C. scoticus*. *Culicoides dewulfi* represented just 3.5% of total catches of all adult forms of *Culicoides* in light-traps but represented 11.8% of the total blood-fed collections made. This disproportionate abundance could be due to host preference towards this host, a tendency for this species to be more actively flying in the blood-fed state than other species, or a greater attraction to light in the blood-fed state than other species, although there is no indication of the latter explanation in studies carried out previously in the UK [31, 54, 55]. Seroconversion to SBV was observed in Asian elephants in the UK previously [18], and this observation demonstrates the potential for transmission of arboviruses to this species. Future studies should look to examine the host preference and utilisation of elephant dung for larval development by *C. dewulfi* as it is one of only two species where larval habitat is considered to be restricted to dung [25, 35].

Similarly, the trap located next to the Bactrian camels at ZSL LZ caught by far the most *Culicoides* at this zoo. Furthermore, two *C. obsoletus/C. scoticus* had fed on the Bactrian camels suggesting that these animals are at a relatively higher risk of vector-borne disease compared to other animals. This is supported by the fact that a single *C. obsoletus/C. scoticus* had been feeding on a Bactrian camel at ZSL WZ, despite them being kept in a large, wind-exposed outdoor paddock, which would be assumed to be less favourable to *Culicoides.* Bactrian camels are susceptible to BTV [56, 57] and a fatal clinical case was identified in a European zoo during the BTV-8 outbreak in northern Europe in 2006 [58].

In addition to susceptible animals, zoos may be home to a number of animals that could act as reservoir hosts for vector-borne diseases. For example, all nine serotypes of AHSV have been isolated from plains zebra in the Republic of South Africa [4] and ZSL LZ has four plains zebra (two *Equus quagga chapmani* and two *Equus quagga burchelli*). Zebra kept in zoos in the UK are unlikely to be of great epidemiological significance, but there may be risk associated with importation of these animals due to their role as reservoir hosts. For example, an outbreak of AHSV occurred in Spain in 1987 due to the importation of zebra from Namibia [4]. The study conducted at Chester Zoo in 2008, concluded that there needs to be pre-import testing of zoo animals arriving to the UK from BTV-endemic areas, due to the potential for onward transmission by UK vectors present at zoos [21]. This was supported by detection of SBV antibodies in three yaks a week after importation from the Netherlands and in a greater kudu, prior to import from France [18].

The seasonal profile for *Culicoides* observed in this study has been previously demonstrated in the UK [31, 59]. These studies have shown peaks in abundance in May/June and again in September and our data also show this bimodal pattern. Variation in the SVFP was driven primarily by two large catches on 16th (*n* = 137 pigmented *Culicoides*) and 30th October (*n* = 63 pigmented *Culicoides*) in the trap AA1 at ZSL LZ. The SVFP ended on 15th April 2015 at both zoos. The mean daily temperature (°C) was higher at ZSL LZ than at ZSL WZ (mean = 12.3 °C and 10.6 °C, respectively, Additional file 3: Figure S1). At a national scale, the SVFP began on 26th November 2014 and ended on 14th April 2015 (M. England, unpublished data). The national scale end date is very close to that found at both zoos, whilst the start date is later at the national scale. This highlights the fact that the measurement of the SVFP is to a significant degree dependent upon the sensitivity of surveillance measures employed. A previous study found that, on average, the end date of the SVFP was early May for years 2006 to 2010 [31]. However, national scale surveillance has reported this date as occurring during April every year from 2014 to 2019, suggesting a trend towards a shorter SVFP in more recent years. Indeed, a recent study identified a long-term shift in abundance and seasonality of *Culicoides* associated with climate change [59]. The significant difference observed in total catch between years is likely to be largely due to the fact that several traps were not operational in 2015 due to damage. Additionally, there were significant differences observed between trap locations for all *Culicoides* species, across both zoos. The greater performance of one trap over another is likely due to a range of factors such as proximity to potential hosts, the level of wind exposure, the relative size and density of hosts surrounding the trap and the availability of larval habitat close to the traps. The dynamics of *Culicoides* at the local scale were examined in a previous study, showing host proximity and exposure were significant factors affecting spatial clustering and abundance [60].

## Conclusions

Zoo animals have a very high value, both financially and as part of international breeding programmes for species conservation. It is, therefore, very important to understand the risk that they face from *Culicoides*-borne arboviruses. Here, we have shown for the first time to our knowledge, through blood-meal analysis, that the putative vectors of SBV and BTV in the UK are feeding on exotic zoo animals. We have also highlighted the need for vaccination and/or mitigating measures for susceptible animals within zoos in the event of an outbreak to protect these endangered species.

## Supplementary information

**Additional file 1: Text S1.** Qiagen DNeasy Blood and Tissue Kit (Qiagen) protocol.

**Additional file 2: Text S2.** Illustra GFX PCR Purification Kit (GE Healthcare) protocol.

**Additional file 3: Figure S1.** Daily mean temperature (°C) for ZSL London Zoo and ZSL Whipsnade Zoo.

**Additional file 4: Dataset 1.** Full data set of collected *Culicoides* including site, trap location, collection date and morphological identification.

**Additional file 5: Table S1.** Species composition of blood-fed *Culicoides* caught in traps.

**Additional file 6: Table S2.** Results of blood meal analysis of *Culicoides* collected from ZSL London Zoo and Whipsnade Zoo.

**Additional file 7: Table S3.** Comparison of different models for the number of *Culicoides* biting midges caught at London and Whipsnade zoos.

**Additional file 8: Table S4.** Effect of seasonality, year, temperature and trap location on the number of *Culicoides* collected.

**Additional file 9: Figure S2.** Observed and expected daily trap catches for *Culicoides obsoletus*/*C. scoticus* females for trap locations at both zoos.

**Additional file 10: Figure S3.** Observed and expected daily trap catches for *Culicoides chiopterus* females for trap locations at both zoos.

**Additional file 11: Figure S4.** Observed and expected daily trap catches for *Culicoides pulicaris* females for trap locations at both zoos.

**Additional file 12: Figure S5.** Observed and expected daily trap catches for *Culicoides punctatus* females for trap locations at both zoos.

**Additional file 13: Figure S6.** Observed and expected daily trap catches for total *Culicoides* for trap locations at both zoos.

## Data Availability

All data generated or analysed during this study are included in this published article and its additional files.
