# Supplementary material for: Culicoides species composition and molecular identification of host blood meals at two zoos in the UK
Source: Parasit Vectors. 2020 Mar 16;13:139. doi: 10.1186/s13071-020-04018-0 (PMC7076997; doi:10.1186/s13071-020-04018-0)
Supplement: Supplementary file 1 — Additional file 1: Text S1. Qiagen DNeasy Blood and Tissue Kit (Qiagen) protocol. [file 13071_2020_4018_MOESM1_ESM.docx]

**Additional file 1: Text S1.**

Qiagen DNeasy Blood and Tissue kit (Qiagen) protocol:

For each sample 200µl of ethanol was added and then mixed by vortexing. The mixture was then pipetted into a spin column in a 2ml collection tube and centrifuged at 8000 rpm for one minute. The flow-through and collection tube was then discarded. The spin column was then transferred to a new 2ml collection tube, 500µl of Buffer AW1 was added and this was then centrifuged at 8000 rpm for one minute. Again, the flow-through and collection tube was discarded. The spin column was again transferred to a new 2ml collection tube, 500µl of Buffer AW2 was added and this was centrifuged at 14 000 rpm for 3 minutes. To elute the DNA, the spin column was placed in a 2ml centrifuge tube and 200µl of Buffer AE was pipetted onto the membrane of the spin column. This was then incubated at room temperature for one minute and then centrifuged at 8000 rpm for one minute. This elution step was repeated once.
