# Supplementary material for: Culicoides species composition and molecular identification of host blood meals at two zoos in the UK
Source: Parasit Vectors. 2020 Mar 16;13:139. doi: 10.1186/s13071-020-04018-0 (PMC7076997; doi:10.1186/s13071-020-04018-0)
Supplement: Supplementary file 2 — Additional file 2: Text S2. Illustra GFX PCR Purification Kit (GE Healthcare) protocol. [file 13071_2020_4018_MOESM2_ESM.docx]

**Additional file 2: Text S2**

Illustra GFX PCR purification kit (GE Healthcare) protocol:

1. Sample Capture
   1. Add 500 µl Capture buffer type 3 to up to 100 µl sample.
   2. Mix thoroughly. Check that the Capture buffer type 3-sample mix is yellow or pale orange in color. For each purification that is to be performed, place one GFX MicroSpin column into one Collection tube.
2. Sample Binding
   1. Centrifuge Capture buffer type 3-sample mix briefly to collect the liquid at the bottom of the tube.
   2. Load the Capture buffer type 3-sample mix onto the assembled GFX MicroSpin column and Collection tube. Note: the cap of the Collection tube can be used to cap the GFX Microspin column. If the cap is not required cut it off.
   3. Spin the assembled column and Collection tube at 16 000 × g for 30 seconds.
   4. Discard the flow through by emptying the Collection tube. Place the GFX MicroSpin column back inside the Collection tube.
3. Wash & Dry
   1. Add 500 µl Wash buffer type 1 to the GFX MicroSpin column.
   2. Spin the assembled column and Collection tube at 16 000 × g for 30 seconds.
   3. Discard the Collection tube and transfer the GFX MicroSpin column to a fresh DNase-free 1.5 ml microcentrifuge tube (supplied by user).
4. Elution
   1. Add 10–50 µl Elution buffer type 4 OR type 6 to the center of the membrane in the assembled GFX MicroSpin column and sample Collection tube.
   2. Incubate the assembled GFX MicroSpin column and sample Collection tube at room temperature for 1 minute.
   3. Spin the assembled column and sample Collection tube at 16 000 × g for 1 minute to recover the purified DNA.
   4. Proceed to downstream application. Store the purified DNA at -20°C.
