# Supplementary material for: Culicoides species composition and molecular identification of host blood meals at two zoos in the UK
Source: Parasit Vectors. 2020 Mar 16;13:139. doi: 10.1186/s13071-020-04018-0 (PMC7076997; doi:10.1186/s13071-020-04018-0)

ZSL London Zoo is represented by the blue line and ZSL Whipsnade Zoo is represented by the red line, from 1st January 2014 to 31st December 2015.


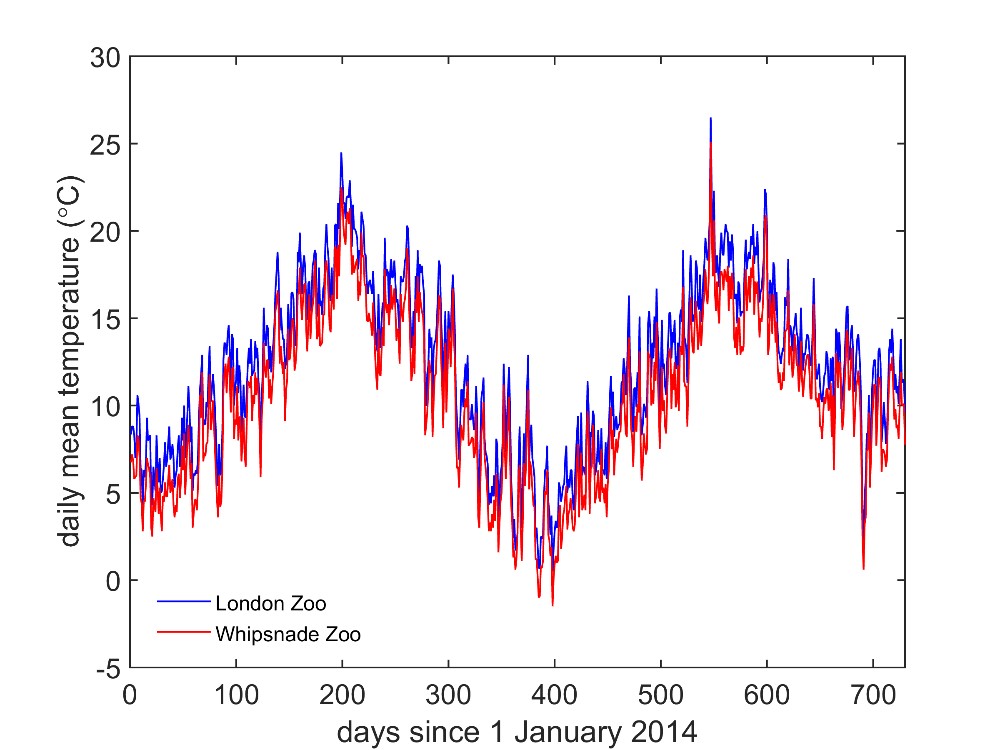

Supplement: Supplementary file 3 — Additional file 3: Figure S1. Daily mean temperature (°C) for ZSL London Zoo and ZSL Whipsnade Zoo. [file 13071_2020_4018_MOESM3_ESM.docx]
