# Supplementary material for: Culicoides species composition and molecular identification of host blood meals at two zoos in the UK
Source: Parasit Vectors. 2020 Mar 16;13:139. doi: 10.1186/s13071-020-04018-0 (PMC7076997; doi:10.1186/s13071-020-04018-0)
Supplement: Supplementary file 12 — Additional file 12: Figure S5. Observed and expected daily trap catches for Culicoides punctatus females for trap locations at both zoos. [file 13071_2020_4018_MOESM12_ESM.docx]

**Additional file 12:** **Figure S5.** Observed and expected daily trap catches for *Culicoides punctatus* females for trap locations at both zoos.


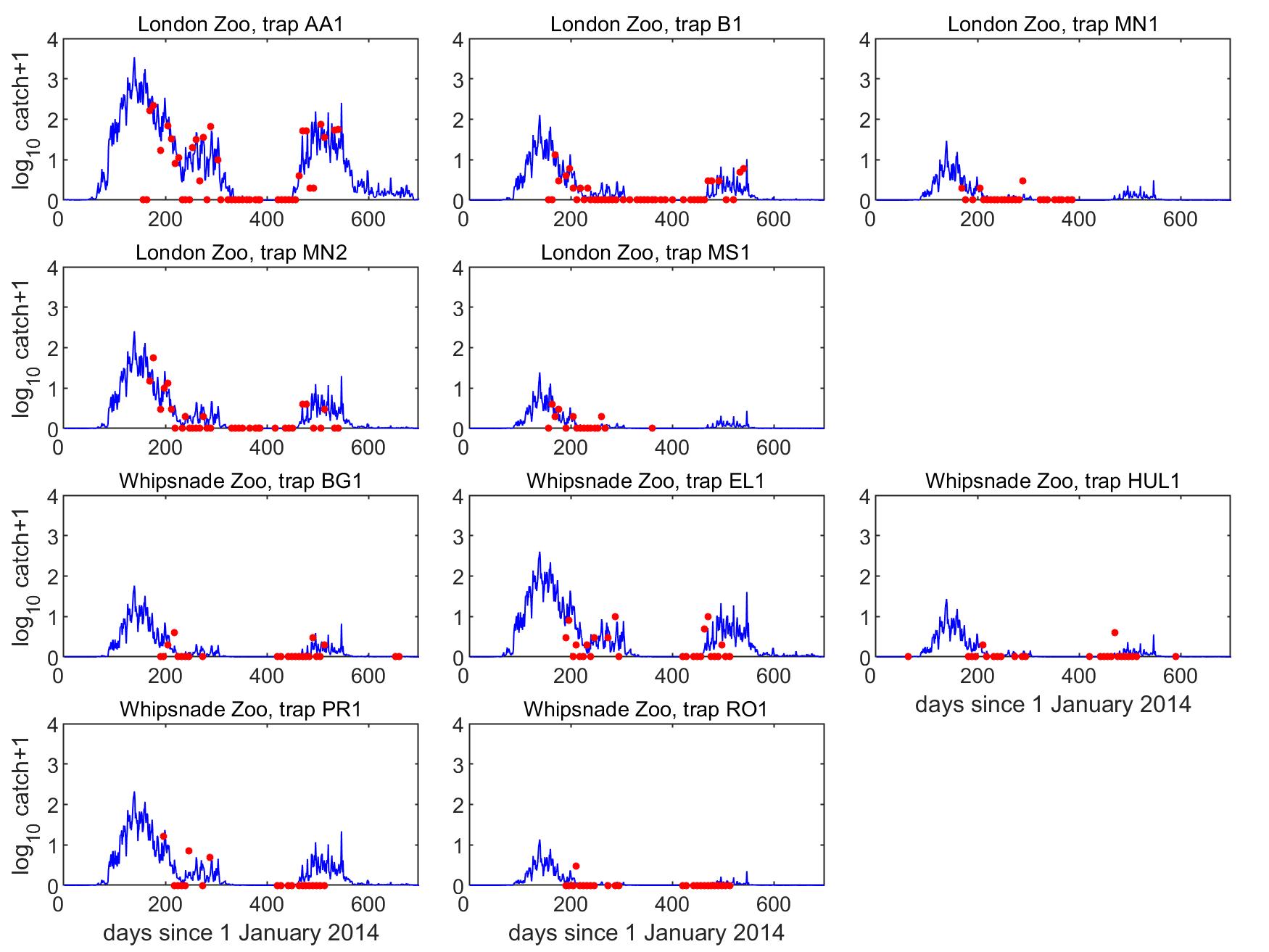


Each plot shows the expected (i.e. the fitted GLM) catch (solid line) and the observed catch (circles) for a trap location.
